# Effects of a brief primary care intervention for post-traumatic stress disorder symptoms after critical illness on health-related quality of life – A secondary analysis of the PICTURE randomised controlled trial

**DOI:** 10.1080/13814788.2026.2702686

**Published:** 2026-07-21

**Authors:** Robert Philipp Kosilek, Nora Schröder, Linda Sanftenberg, Caroline Jung-Sievers, Daniela Lindemann, Antina Beutel, Konrad Schmidt, Christian Brettschneider, Hans-Helmut König, Thomas Elbert, Jochen Gensichen

**Affiliations:** a Institute of General Practice and Family Medicine, LMU University Hospital, LMU Munich, Munich, Germany; b Institute for Medical Information Processing, Biometry, and Epidemiology - IBE, Chair of Public Health and Health Services Research, LMU Munich, Munich, Germany; c Pettenkofer School of Public Health, Munich, Germany; d Department of Psychology, University of Konstanz, Konstanz, Germany; e Institute of General Practice and Family Medicine, Charité University Medicine, Berlin, Germany; f Institute of General Practice, Faculty of Health Sciences Brandenburg, Brandenburg Medical School Theodor Fontane, Brandenburg, Germany; g Department of Health Economics and Health Services Research, University Medical Center Hamburg-Eppendorf, Hamburg, Germany; h German Center for Mental Health (DZPG), Munich, Germany

**Keywords:** Critical illness, intensive care units, post-traumatic stress disorders, health-related quality of life, patient-reported outcomes

## Abstract

**Background:**

Intensive care unit (ICU) survivors frequently experience symptoms of post-traumatic stress disorder (PTSD) and reduced health-related quality of life (HRQoL), yet evidence for effective post-ICU interventions in primary care remains limited. This secondary analysis of the PICTURE randomised controlled trial provides an in-depth evaluation of HRQoL trajectories and potential treatment mechanisms.

**Methods:**

The analysis included 319 adult ICU survivors with at least moderate PTSD symptoms, randomised to a brief general practitioner (GP)-delivered narrative exposure intervention or enhanced usual care. Longitudinal mixed-effects models estimated treatment-by-time effects on HRQoL, based on EuroQol Five-Dimension Five-Level (EQ-5D-5L) index and visual analog scale (VAS), with mediation *via* PTSD symptom change assessed using structural equation modelling.

**Results:**

The mean baseline EQ index was 0.71 ± 0.27 and EQ VAS was 60.7 ± 19.4. Mixed-effects models showed a transient improvement in EQ VAS at 6 months in the intervention group (ß = 5.85; 95% CI 0.84–10.87), followed by a delayed improvement in the EQ index at 12 months (ß = 0.075; 95% CI 0.014–0.136). About one quarter of the 12-month EQ index effect was mediated by PTSD symptom reduction at 6 months. Domain-level analyses indicated greater improvement in anxiety/depression and mobility.

**Conclusions:**

In ICU survivors with PTSD symptoms, a brief GP-delivered psychological intervention was associated with clinically meaningful improvements in health-related quality of life over 12 months, with effects emerging over time and only partly explained by PTSD symptom reduction.

**Trial Registration:**

ClinTrials.gov: NCT03315390 (Registration date: 2017-10-20); German Clinical Trials Register (DRKS): DRKS00012589 (Registration date: 2017-10-17)

## Background

Most follow-up after treatment on an intensive care unit (ICU) takes place in primary care, where general practitioners (GPs) increasingly treat survivors of critical illness and are advised to screen for depression, anxiety, post-traumatic stress disorder (PTSD) and cognitive impairment [1,2]. Mental health problems are common in this population. During the first year after leaving the ICU, roughly one in five survivors develop symptoms of PTSD and around one in three report significant anxiety or depressive symptoms [3–5]. These disturbances are closely intertwined with reduced health-related quality of life (HRQoL) and difficulties resuming previous social and occupational roles [6,7]. Despite this, structured post-ICU programs and evidence-based psychological interventions remain scarce, and access to specialist mental health care is often delayed [8]. Trials of ICU follow-up clinics and rehabilitation interventions have produced inconsistent results for functional outcomes and HRQoL, leaving GPs with limited guidance on how best to support recovery in this group [9–11]. These challenges are rooted in the broader syndrome known as post-intensive care syndrome (PICS), which encompasses new or worsened physical, mental and cognitive impairments following critical illness [12,13]. There is growing recognition that patient-important outcomes such as physical function, psychological well-being and social reintegration should be routinely assessed [14–16]. Brief psychological interventions that can be delivered within primary care are a promising way to address this unmet need. Narrative exposure therapy (NET) is a short, trauma-focused treatment that has demonstrated efficacy when delivered by non-specialist health-care professionals [17]. The PICTURE trial evaluated a GP-led brief NET intervention for ICU survivors and demonstrated favourable effects on PTSD symptoms as the primary outcome [18]. Building on these findings, the present work is an exploratory secondary analysis of HRQoL. It characterises 12-month trajectories and examines potential treatment mechanisms by assessing whether HRQoL changes are mediated by earlier improvements in PTSD symptoms. In addition, the observed effects are contextualised within the broader HRQoL literature, providing a detailed assessment of whether observed changes correspond to clinically meaningful, patient-important gains in a primary-care based psychological intervention.

## Methods

### Study design and population

This analysis uses data from the multicentre PICTURE randomised controlled trial, conducted in German primary care between 2018 and 2022 to test a brief PTSD intervention based on NET. Here we report a detailed longitudinal intention-to-treat analysis of HRQoL as a predefined secondary outcome. Adults aged 18–85 years were recruited *via* collaborating hospitals and general practices if they were ICU survivors of significant critical illness (respiratory support, sequential organ failure assessment – SOFA score ≥ 3) and had at least moderate PTSD symptoms (PDS-5 15–70); patients with severe physical or mental health conditions that precluded consent or follow-up were excluded. In total, 319 eligible participants provided informed consent and completed the baseline assessment. Eligible GPs were community-based and had basic psychosomatic care training or an equivalent qualification, and were reimbursed in line with standard insurance tariffs for psychosomatic consultations. The trial was approved by the ethics committee at LMU Munich (#17–436) and conducted in accordance with the Declaration of Helsinki, and is described in detail in the published protocol and primary outcome reports [18,19].

### Intervention

Participants were randomised 1:1 by a computer-generated sequence to intervention or control at the level of patient-GP dyads, with each pair allocated exclusively to one study arm. The intervention comprised three GP-delivered narrative exposure sessions over about six weeks, supported by eight scheduled practice nurse contacts. Sessions followed an adapted NET protocol, in which the traumatic critical illness event was reconstructed and integrated into the patient’s autobiographical memory, while nurses provided case management and care coordination. Controls received enhanced usual care based on the German PTSD guideline: GPs were asked to deliver guideline-concordant care across three consultations but were not trained in NET. In both groups, use of standard health services and specialist referrals was unrestricted, but concomitant trauma-specific psychotherapy was an exclusion criterion.

### Outcomes and measurements

HRQoL was assessed with the EuroQol EQ-5D-5L questionnaire, covering five dimensions (mobility, self-care, usual activities, pain/discomfort, anxiety/depression), each rated on a five-level scale from 1 to 5, with higher values indicating greater problems (worse health status) [20]. It yields an index score, reflecting health across these domains weighted by societal preferences, derived using the German value set [21], ranging from ‘worse than death’ (–0.661) through 0 (death) to 1 (full health). It additionally contains a visual analog scale (VAS) ranging from 0 (worst imaginable health) to 100 (best imaginable health), reflecting overall self-rated health. Baseline covariates were obtained *via* structured interview and chart review, including age, sex, and education (assessed using the CASMIN classification of educational attainment), as well as ICU-related variables (primary ICD-10 diagnosis, length of stay, Sequential Organ Failure Assessment – SOFA – Score). PTSD symptoms were measured with the Post-traumatic Diagnostic Scale for DSM-5 (PDS-5), comprising 20 items plus four supplementary questions (range 0–80 points, ≥36 indicating probable PTSD) [22,23].

### Statistical analysis

Analyses were performed in Stata 19.0 (StataCorp, College Station, TX, USA). Baseline characteristics and EQ-5D-5L measures including crude changes over time were summarised descriptively. Primary longitudinal analyses for EQ index, VAS and domains used mixed-effects linear regression models with random intercepts for individuals to account for repeated measures over time, including fixed effects for treatment, time, and their interaction. We estimated unadjusted models and models adjusted for age, sex, education (high vs. intermediate/low), ICU index diagnosis (cardiovascular disease vs. other), and baseline PDS-5 scores. To address potential attrition bias, adjusted models were additionally weighted using stabilised inverse probability weights (SIPW) derived from logistic models for retention at 6 and 12 months conditional on treatment, sociodemographic variables, ICU diagnosis, and baseline EQ index and PDS-5 scores; the resulting weights had a mean of 1.0 (SD 0.37) with no extreme values. Trajectories were visualised using marginal effects plots of model-based means across baseline, 6-month, and 12-month follow-up for both groups. To account for ceiling effects in the EQ index in a sensitivity analysis, EQ disutility (1-index) was additionally modelled using a two-part model (logit model for any disutility and gamma model for positive disutility) fit separately at 6 and 12 months, weighted and adjusted for the same covariate set (including baseline EQ disutility), with results combined as marginal effects [24]. To examine mechanisms, a structural equation model assessed whether changes in PTSD symptoms at 6 months mediated treatment effects on EQ index at 12 months. Direct, indirect, and total effects were derived using the product-of-coefficients approach with delta-method confidence intervals. Models were estimated using maximum likelihood with missing values. Heteroskedasticity-robust standard errors were applied throughout [25].

## Results

Among 319 participants (mean age 57.7 years ± SD 12.7; 60.8% male), cardiovascular disease was the leading ICU diagnosis (40.4%). The median ICU stay was 8.0 days (IQR 4.0–18.0) with a mean SOFA score of 9.5 ± 3.9; 30.1% were emergency admissions. PTSD symptom severity was overall moderate (PDS-5 mean 30.6 ± 13.3). The EQ-5D-5L index mean was 0.71 ± 0.27; the EQ VAS mean was 60.7 ± 19.4. Mean EQ-5D-5L domain scores were 2.04 ± 1.20 for mobility, 1.50 ± 0.95 for self-care, 2.17 ± 1.18 for usual activities, 2.40 ± 1.06 for pain/discomfort, and 2.22 ± 1.06 for anxiety/depression. Characteristics were balanced except for higher education in controls. Full baseline characteristics are shown in Table 1. Follow-up was 84.9% (*n* = 271) at 6 months and 77.4% (*n* = 247) at 12 months. GPs were 50.5% male, with a mean patient relationship 6.8 ± 7.4 years; most were family physicians (62.7%), working in group practices (52.4%) and urban areas (40.1%).

**Table 1.** Baseline cohort profile by randomisation status.

	Control	Treatment	Total
	*N* = 159	*N* = 160	*N* = 319
Gender (male), % (No.)	64.2% (102)	57.5% (92)	60.8% (194)
Age (years), mean (SD)	57.6 (13.2)	57.8 (12.2)	57.7 (12.7)
Education (CASMIN levels), % (No.)			
*Low (1a-1c)*	*27.0% (43)*	*25.6% (41)*	26.3% (84)
*Intermediate (2a-2c)*	*35.2% (56)*	*49.4% (79)*	42.3% (135)
*High (3a-3b)*	*32.1% (51)*	*21.2% (34)*	26.6% (85)
*N/A*	*5.7% (9)*	*3.8% (6)*	4.7% (15)
Main ICU diagnosis (ICD-10), % (No.)			
*I (Cardiovascular disease)*	*42.8% (68)*	*38.1% (61)*	40.4% (129)
*J (Respiratory disease)*	*11.9% (19)*	*15.6% (25)*	13.8% (44)
*U (Other: COVID-19)*	*7.5% (12)*	*6.9% (11)*	7.2% (23)
*C (Neoplasms)*	*5.7% (9)*	*8.1% (13)*	6.9% (22)
*K (Gastrointestinal disease)*	*2.5% (4)*	*9.4% (15)*	6.0% (19)
*Other*	*29.6% (47)*	*21.9% (35)*	25.7% (82)
Emergency admission, % (No.)	32.1% (51)	28.1% (45)	30.1% (96)
ICU stay (days) (*N* = 312), median (IQR)	7.0 (4.0–18.0)	8.0 (4.0–18.0)	8.0 (4.0–18.0)
SOFA score (*N* = 270), mean (SD)	9.6 (3.7)	9.4 (4.1)	9.5 (3.9)
PDS-5 score, mean (SD)	30.7 (13.2)	30.4 (13.4)	30.6 (13.3)
EQ-5D-5L VAS (*N* = 318), mean (SD)	60.5 (18.9)	61.0 (19.9)	60.7 (19.4)
EQ-5D-5L index, mean (SD)	0.72 (0.28)	0.71 (0.27)	0.71 (0.27)

CASMIN: Comparative Analysis of Social Mobility in Industrial Nations classification; ICU: intensive care unit; ICD-10: International Classification of Diseases, 10th Revision; SOFA: Sequential Organ Failure Assessment; PDS-5: Posttraumatic Diagnostic Scale for DSM-5; EQ-5D-5L: EuroQol five-dimension five-level questionnaire; EQ VAS: EuroQol visual analogue scale; EQ index: EQ-5D utility index score. Higher PDS-5 scores indicate greater PTSD symptom burden, whereas higher EQ index and EQ VAS scores indicate better health-related quality of life.

**Figure 1.**
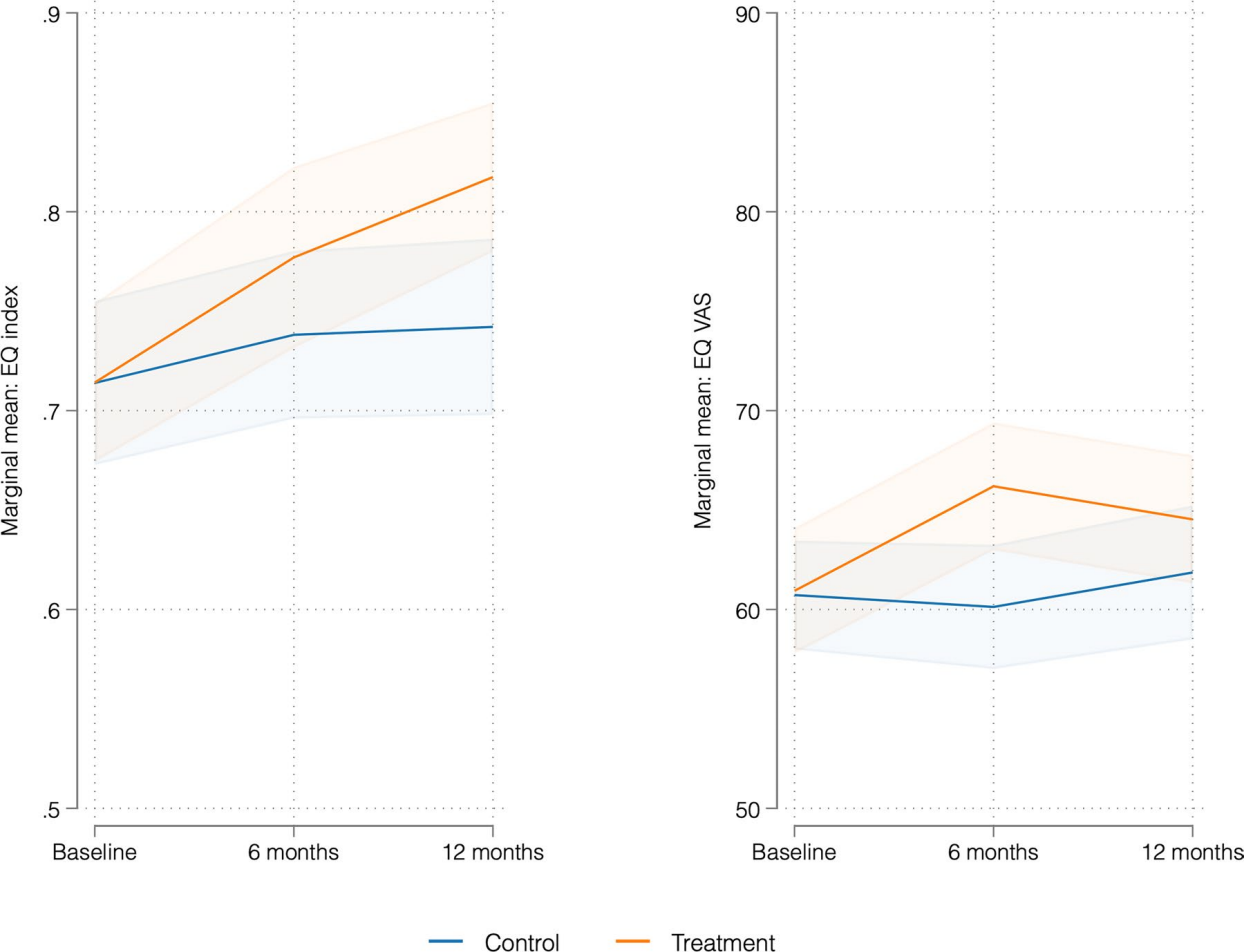
EQ-5D-5L index and VAS trajectories by treatment group over time. Trajectories represent adjusted marginal means derived from mixed-effects linear regression models for EQ index (*n* = 319) and EQ VAS (*n* = 318) at baseline, 6 months, and 12 months. EQ-5D-5L: EuroQol five-dimension five-level questionnaire; EQ index: EQ-5D-5L utility index score (range −0.661 to 1, higher scores indicating better health); EQ VAS: EuroQol visual analogue scale (0–100, higher scores indicating better health).

For the EQ index, the crude between-group difference was 0.037 (*p* = 0.223) at 6 months (*n* = 263) and 0.091 (*p* = 0.004) at 12 months (*n* = 245); the respective between-group differences for the EQ VAS were 5.00 (*p* = 0.051) and 2.39 (*p* = 0.390). Mixed-effects models used 827 observations from 319 individuals (EQ index) and 826 observations from 318 individuals (EQ VAS) (Figure 1). The intraclass correlation coefficient was 0.54 for the EQ index and 0.38 for EQ VAS. Baseline values were comparable between groups; controls showed minimal change over time. Treatment effects were estimated *via* treatment-by-time interactions. For the EQ index, no clear effect was observed at six months (*β* = 0.039 [95% confidence inverval: −0.022 to 0.100]), while a significant improvement emerged at twelve months (*β* = 0.075 [0.014 to 0.136]). For the EQ VAS, a transient effect was observed at six months (*β* = 5.85 [0.84 to 10.87]), which was not sustained at twelve months (*β* = 2.46 [−2.84 to 7.75]). Models were adjusted for prespecified covariates (age, sex, high education, cardiovascular diagnoses, baseline PTSD symptoms) and weighted using inverse probability weights to account for differential drop-out. Adjustment did not materially alter the treatment effect estimates, which were similar in unadjusted models including only the treatment-by-time interaction (EQ index: 0.032 [−0.027 to 0.091] and 0.073 [0.013 to 0.133]; EQ VAS: 5.48 [0.60–10.36] and 2.00 [−3.24 to 7.23]; at 6 and 12 months, respectively). Additional sensitivity analyses using adjusted two-part models for EQ-5D disutility (1 − index) yielded comparable results for the treatment effects (6 months: *β* − 0.045 [−0.101 to 0.011]; 12 months: *β* − 0.087 [−0.141 to −0.032]).

**Figure 2.**
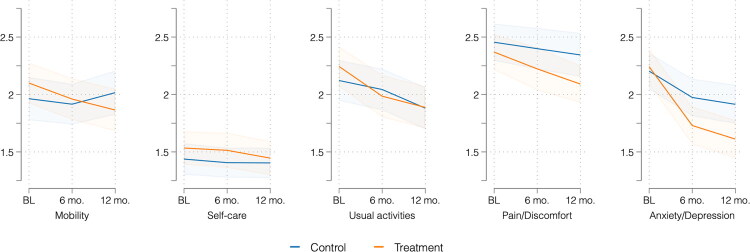
EQ-5D-5L domain trajectories by treatment group over time. Trajectories represent adjusted marginal means derived from mixed-effects linear regression models for EQ-5D-5L domains (*n* = 319) at baseline, 6 months, and 12 months. EQ-5D-5L: EuroQol five-dimension five-level questionnaire; domain scores range from 1 to 5, with higher values indicating greater impairment in the respective domain.

In adjusted mixed-effects models for the EQ-5D-5L domains (Figure 2), the intervention showed significant reductions in anxiety/depression at both 6 months (*β* = −0.28 [−0.56 to −0.01]) and 12 months (*β* = −0.34 [−0.63 to −0.05]). For mobility, a significant improvement emerged at 12 months (*β* = −0.29 [−0.54 to −0.03]), with no clear effect at 6 months (ß = −0.09 [−0.33 to 0.15]). In contrast, effects for self-care (6 months: ß = 0.01 [−0.16 to 0.19]; 12 months: ß = −0.05 [−0.25 to 0.14]), usual activities (−0.18 [−0.45 to 0.09]; −0.12 [−0.40 to 0.17]), and pain/discomfort (−0.09 [−0.35 to 0.17]; −0.17 [−0.44 to 0.10]) were smaller and not statistically significant.

**Figure 3.**
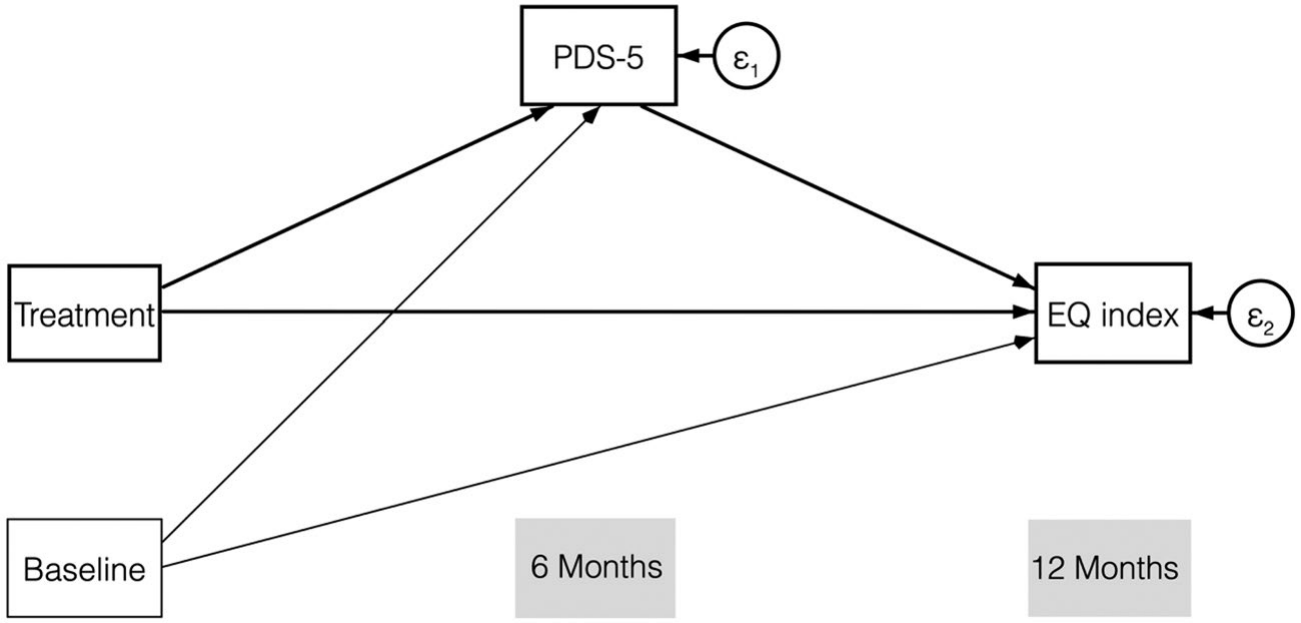
Mediation model. Simplified mediation model: randomised treatment and baseline covariates predict 6-month PTSD symptoms (PDS-5) and 12-month EQ index, with direct and indirect pathways to EQ index and residual variances ε_1_ and ε_2_, implemented via structural equation modelling (Table 2). PDS-5: Posttraumatic Diagnostic Scale for DSM-5; EQ index: EuroQol five-dimension five-level utility index score.

In the mediation analysis (Table 2; Figure 3), the treatment reduced PDS-5 scores at 6 months (*β* = −4.81 [−7.72 to −1.91]). Higher PDS-5 scores at 6 months predicted lower 12-month EQ index (*β* = −0.004 [−0.006 to −0.002]). The direct treatment effect on the 12-month EQ index was 0.060 [0.009–0.110], with an indirect effect *via* 6-month PDS-5 of 0.020 [0.005–0.035], yielding a total effect of 0.080 [0.029–0.131]. Approximately one quarter of the 12-month EQ index effect (ratio indirect/total 0.25) was mediated by earlier improvements in PTSD symptoms.

**Table 2.** EQ index at 12 months: mediation model.

	Equation 1: PDS-5 at 6 months		Equation 2: EQ index at 12 months	
**Direct effects**	ß	95% CI	ß	95% CI
Treatment	−4.39***	[−7.28 to −1.50]	0.060**	[0.008–0.111]
PDS-5 score at 6 months	–	–	−0.004***	[−0.007 to −0.002]
PDS-5 score at baseline	0.69***	[0.58–0.81]	0.002	[−0.001 to 0.005]
EQ index at baseline	−6.10**	[−11.95 to −0.25]	0.434***	[0.314–0.553]
Age (years)	−0.09**	[−0.21 to 0.03]	−0.002**	[−0.004 to −0.000]
Gender (male)	1.30	[−1.72 to 4.32]	0.024	[−0.030 to 0.078]
Education (high)	−1.49	[−4.54 to 1.57]	0.062***	[0.015–0.109]
ICU diagnosis (ICD-10: I.x)	−3.05*	[−6.21 to 0.12]	0.019	[−0.032 to 0.069]
Intercept	18.39		0.584	
Residual variance	141.73		0.039	
R^2^	0.43		0.38	
**Indirect effects**				
Treatment *via* PDS-5 at 6 months			0.020***	[0.004–0.035]
**Total effects**				
Treatment			0.079***	[0.027–0.131]
*Ratio indirect/total*			*0.25*	

PDS-5: Posttraumatic Diagnostic Scale for DSM-5; EQ index: EuroQol five-dimension five-level utility index score; ICU: intensive care unit; ICD-10: International Classification of Diseases, 10th Revision. Higher PDS-5 scores indicate greater PTSD symptom burden, whereas higher EQ index scores indicate better health-related quality of life.

*N* = 319. Structural equation model with two equations: (1) PDS-5 score at 6 months regressed on treatment and covariates, and (2) EQ-5D-5L index at 12 months regressed on treatment, 6-month PDS-5, and covariates. Models were estimated using full information maximum likelihood (FIML) with robust standard errors and stabilised inverse probability weights for attrition adjustment. Indirect, direct, and total effects were derived using the product-of-coefficients approach.

*** *p < 0.001*, **p < 0.01, *p < 0.05.

## Discussion

### Main findings

This secondary analysis of the PICTURE trial examined EQ-5D-5L trajectories and treamentment effects of a brief GP-led narrative exposure intervention for ICU survivors with PTSD symptoms. Baseline EQ index and VAS scores indicated substantial impairment compared with German general population norms [26]. The intervention arm demonstrated better self-rated health at 6 months and higher utility-based HRQoL at 12 months, which was partly mediated through earlier reductions in PTSD symptoms at 6 months, and gains mainly in the anxiety/depression and mobility domains.

#### Comparison with existing literature

These findings extend prior evidence on post-ICU morbidity, which has consistently documented persistent physical and psychological impairments and associated reductions in HRQoL [27,28], while trials of ICU follow-up and rehabilitation shown mixed effects [9–11]. A systematic review reports minimally important difference (MIDs) for the EQ-5D-5L index and EQ VAS of 0.065 (IQR 0.057) and 9.0 (IQR 5.0), respectively [29]. Against this background, the total 12-month treatment effect of approximately +0.08 on the EQ index represents a clinically meaningful improvement in HRQoL. The mediation analysis suggests that roughly one quarter of the EQ index improvement is attributable to earlier reductions in PTSD symptoms, indicating that symptom change contributes to – but does not fully account for – subsequent HRQoL gains. The remaining treatment effect likely reflects spill-over benefits into mental and functional health domains not captured by the PDS-5, such as gradual psychological adjustment and re-engagement in daily roles. Features characteristic of GP-led care, such as continuity and patient–physician familiarity, may have been further intensified by the intervention – potentially through longer consultations, closer interaction, and more structured follow-up – and thereby contributed to enhanced recovery processes. Temporal patterns indicate a staged trajectory: A transient improvement in EQ VAS at 6 months suggests early gains in subjective health perception, whereas improvements in the EQ index emerged at 12 months. Preference-based index scores may respond more slowly due to domain aggregation and ceiling effects, whereas visual analogue ratings may capture earlier, more flexible perceptions of health status. At 12 months, the clearest domain-specific improvements were observed in anxiety/depression and mobility, consistent with a trauma-focused intervention targeting emotional distress, potentially reducing related functional limitations reflected in improved mobility.

### Strengths and limitations

Strenghts of this study include its randomised controlled design in routine primary care, a clearly defined group of ICU survivors with at least moderate PTSD symptoms, and longitudinal assessment using validated instruments over 12 months. Several limitations should be considered. HRQoL was a secondary outcome, and the trial was not powered to detect small differences in the EQ-5D-5L instrument. The mediation analysis, although based on randomisation and temporal ordering, is observational for the mediator–outcome pathway and cannot establish causality; it should be interpreted as exploratory evidence of a potential pathway rather than confirmatory proof of mechanism. Although we use longitudinal maximum likelihood estimation complemented by inverse probability weighting for dropout adjustment, residual bias due to unobserved missingness mechanisms cannot be fully excluded. Reliance on self-report mental health scales may have introduced reporting bias. The control condition represented an enhanced usual care approach, which may have reduced between-group contrast compared with standard usual care, potentially attenuating observed effect estimates. Finally, the sample selected for psychological distress and conduct in German primary care may limit generalisability to other ICU populations and health systems.

### Implications for research, clinical practice, education, or policy

The findings have implications for practice and research. Post-ICU care in Germany is not delivered through a structured pathway but is mainly managed in general practice, with access to specialist mental health services often constrained by long waiting times [8]. The intervention was designed for this setting and showed high participant adherence and low GP dropout in the main trial, supporting its feasibility in routine care. Its brief, manualised format and limited training requirements make it suitable for primary care. Although the trial did not reach the pre-specified MCID of 6 points on the PDS-5 at 6 months, the observed EQ index improvement over 12 months exceeded established thresholds for clinical importance, suggesting that even modest reductions in PTSD symptoms can translate into meaningful gains in overall health status. Even modest HRQoL gains, particularly in psychological domains, may be meaningful for patients with long-term sequelae [30]. In a primary care context, where interventions are typically lower intensity and embedded in routine care, these findings support further investigation into mechanisms through which such approaches generate patient-important benefits beyond symptom reduction alone. While implementation in other healthcare systems may depend on differences in organisation and access to specialist care, the approach appears broadly transferable to similar GP-led contexts.

## Conclusion

In summary, this secondary analysis of the PICTURE trial shows that a brief GP-delivered narrative exposure intervention in ICU survivors with PTSD symptoms is associated with clinically meaningful improvements in health-related quality of life. The benefit follows a time-dependent pattern, with early gains in self-rated health and subsequent improvements in preference-based utility at 12 months, partly explained by earlier reductions in PTSD symptom burden. These findings indicate that changes in HRQoL are not solely driven by PTSD symptom reduction and support further evaluation of brief psychological interventions in post-ICU follow-up, particularly compared with more intensive or multidisciplinary rehabilitation.

## Data Availability

The analytical dataset, which includes de-identified patient data, is available in the research data repository of the Ludwig-Maximilians-University of Munich ‘Open Data LMU’ and can be accessed at https://data.ub.uni-muenchen.de/557/. Access to the dataset is subject to our data use agreement, and further details can be found in the repository documentation. For enquiries about data use, potential collaborations or related projects, interested researchers are encouraged to contact the principal investigator of the study.

## The PICTURE study group, in alphabetical order

*Christine Adrion, Matthias Angstwurm, Antje Bergmann, Antina Beutel, Gerhard Bielmeier, Andrea Bischhoff, Ralph Bogdanski, Katja Brenk-Franz, Franz Brettner, Christian Brettschneider, Josef Briegel, Martin Bürkle, Johanna Dohmann, Thomas Elbert, Peter Falkai, Thomas Felbinger, Richard Fisch, Hans Förstl, Benjamin Fohr, Martin Franz, Patrick Friederich, Chris-Maria Friemel, Jürgen Gallinat, Sabine Gehrke-Beck, Jochen Gensichen, Herwig Gerlach, Andreas Güldner, Hanna Hardt, Irene Heimbeck, Christoph Heintze, Andreas Heinz, Axel Heller, Simona Hennig, Christian von Heymann, Petra Hoppmann, Volker Huge, Michael Irlbeck, Ulrich Jaschinski, Dominik Jarczak, Stefanie Joos, Elisabeth Kaiser, Melanie Kerinn, Frank-Rainer Klefisch, Stefan Kluge, Roland Koch, Thea Koch, Hans-Helmut König, Robert Philipp Kosilek, Michelle Kowalski, Peter Lackermeier, Karl-Ludwig Laugwitz, Tri Le, Yvonne Lemke, Achim Lies, Klaus Linde, Daniela Lindemann, Dagmar Lühmann, Stephanie May, Ludwig Ney, Jan Oltrogge, Wulf Pankow, Sergi Papiol, Maximilian Ragaller, Nikolaus Rank, Jonas Raub, Lorenz Reill, Ulf-Dietrich Reips, Siegfried Reitberger, Hans-Peter Richter, Reimer Riessen, Grit Ringeis, Ann Rüchardt, Linda Sanftenberg, Maggie Schauer, Gustav Schelling, Jörg Schelling, André Scherag, Martin Scherer, Konrad FR Schmidt, Antonius Schneider, Gerhard Schneider, Jürgen Schneider, Sophie Schneider, Julia Schnurr, Nora Schröder, Tomke Schubert, Susanne Schultz, Thomas G Schulze, Karin Schumacher, John Singhammer, Peter Spieth, Kerstin Theisen, Franka Thurm, Natalia Unruh, Thomas Vogl, Karen Voigt, Cornelia Wäscher, Andreas Walther, Dietmar Wassilowsky, Regina Wehrstedt, Roland Weierstall-Pust, Marion Weis, Björn Weiss, Georg Weiss, Harald Well, Christian Zöllner, Bernhard Zwissler.*
